# Effects of Dance Interventions on Aspects of the Participants' Self: A Systematic Review

**DOI:** 10.3389/fpsyg.2018.01130

**Published:** 2018-07-17

**Authors:** Tina M. Schwender, Sarah Spengler, Christina Oedl, Filip Mess

**Affiliations:** Department of Sport and Health Sciences, Technical University of Munich, Munich, Germany

**Keywords:** dance, self, intervention, systematic review, children, adolescents, adults

## Abstract

**Background:** Theoretical and empirical studies indicate that dance can strengthen the participants' self. The aim of the systematic review is to give an overview of studies investigating the effects of dance interventions on aspects of the self (e.g., self-concept/-esteem). Research questions are: (a) What is the evidence of the reported effects on different aspects of the self in children/adolescents and in adults? (b) Which study approaches and designs are used and what characterizes the interventions? (c) What are the qualitative facets of the implemented studies and what are issues for future research?

**Methods:** We searched online databases for English and German journal articles with the following main inclusion criteria: (i) Intervention study (qualitative and quantitative approaches) (ii) Investigation of aspects of the self (iii) Dance as intervention content. Two reviewers independently screened studies for eligibility using the PRISMA guidelines and assessed the methodological quality of the included studies.

**Results:** Out of 24 included studies, 11 investigate a sample of children/adolescents and 13 an adult sample. The review showed that dance interventions can have positive effects on aspects of the participants' self. The review of studies with qualitative methodologies suggests: children/adolescents benefit in body-related perceptions, self-trust, self-esteem, self-expression and perception of dance-abilities; adults benefit in self-expression, self-efficacy, self-/body-awareness, self-development and self-confidence. Studies with quantitative methodologies report improvement especially for body-related perceptions in both populations. Contradictory results exist concerning self-esteem/-efficacy. The evaluated studies show a heterogeneous nature of populations, intervention contents, timeframes, outcomes, research methods and study quality. Evidence for each of the aspects is still poor due to the small number of studies on each construct, inconsistent findings or methodological shortcomings.

**Conclusions:** This review indicates that dance may be a valuable approach to strengthen aspects of the self. However, as evidence for the different aspects of the self is still poor, further studies with high quality are required (e.g., large samples, active control group). Research considering the complexity and specificity of dance interventions in the design and reporting (e.g., choice of outcomes, presentation of intervention details) seem to be particularly suitable to capture the effects of dance considering its holistic nature.

## Introduction

Representatives of the dance field emphasize the value of dance movement compared to movement in sports or in daily routine (Laban, 1948; Haselbach, 1976; Fritsch, 1988; Peter-Bolaender, 1991). Unlike normative sports, dance offers the opportunity to integrate the variety of movement possibilities. Moreover, dance movement differs from most movements in sports and daily routine regarding internal and external orientation: according to Ullmann, in dance the moving person immerses in the process of moving themself without paying attention to the practical result of the movement action (Ullmann, 2001). Gurley et al. also emphasize its difference with respect to goal-oriented or competitive aspects of sports movement and point out the expressive, creative and aesthetic character of dance movement (Gurley et al., 1984). Dance gives the opportunity to involve different senses and connects movement to music with self-expression; therefore, it is seen as activity that addresses various facets of the personality (Kirsch, 2005; Kaufmann, 2011; Studer-Lüthi and Züger, 2012). In accord with this presumption, especially representatives of the dance education value dance as a specific opportunity to raise self- and body-awareness (Fritsch, 1988; Kirsch, 2005). Rudolf von Laban's work *Modern Educational Dance* pursues the development of the individual and substantially influences dance teachers' work to this day (Laban, 1948). The aim of dance education in Laban's tradition is neither artistic nor technical perfection but the beneficial effect of the creative activity upon the personality of the individual at any age. As these theoretical explanations indicate that dance has the potential to especially strengthen aspects of the self, this review intends to focus on this psychological construct.

*The self*, its' complexity and structure is the subject of different scientific disciplines like educational theory and psychology (Asendorpf and Neyer, 2012; Peichl, 2013). Baumeister defines the self as the totality of a person including the physical self and the self that is constructed out of meaning (Baumeister, 1991). Schuetz outlines two components of the self (Schütz, 2003): it encompasses self-concept as the cognitive-descriptive part and self-esteem as the emotional-affective component. Self-esteem results from the subjective evaluations of the self-related knowledge accumulated in self-concept. The superordinate term self-concept implies a person's self-perceptions and is essential to approach the question “Who am I?” (Amelang et al., 2011). Current research (Marsh and Hattie, 2011) still emphasizes the multidimensional, hierarchical model of self-concept proposed by Marsh and Shavelson (1985). Marsh and Hattie point out that this hypothetical construct could be valuable in explaining a person's behavior (Marsh and Hattie, 2011). Shaped by the experiences with the individual surroundings and by the interpretation and evaluation of the same, it functions as both outcome and mediator. According to Amelang, the expectations of self-efficacy are included in self-concept; the expectations of self-efficacy are defined as a person's beliefs in their competencies and their ability to practice them in adequate situations (Amelang et al., 2011). The fact that self-perceptions influence a person's behavior explains their relevance.

According to Block and Kissel, the interacting system of body and mind is reflected in the holistic activity of dancing: the authors see dance as “essence of embodiment,” as dance expresses the meaning of an embodied being-in-the-world (Block and Kissell, 2001). The holistic idea of an interaction of body and mind corresponds to the *embodiment theory*. Following the embodiment theory there are interplays of mind, body and environment (Tschacher and Bergomi, 2011). Thereby human mind is seen as embedded in the body, which, in turn, is integrated into the environment. Koch stresses the bidirectionality assumption between the motor system and the cognitive-affective system (Koch, 2011). Consequently, motor behavior and body movements can be both the reason for and effect of psychological experience (Storch et al., 2011). Results in the experimental field of movement feedback support the direction of action in which movement or body posture impact a person's affect, attitude and perception (Koch, 2011; Storch et al., 2011). Hence, it seems reasonable that psychological constructs are influenced in the process of dancing.

*Current literature* offers reviews that point out the effects of dance interventions. For example, Burkhardt and Brennan conducted a review of controlled trials on the effects of recreational dance interventions on the health and wellbeing of children and young people (Burkhardt and Brennan, 2012). They report that recreational dance can improve cardiovascular fitness and bone health and can contribute to preventing or reducing obesity. They also refer to some studies that find positive effects on the participants' self-concept, body image and anxiety. However, especially for the psychosocial findings they state limited evidence. Moreover, a number of reviews exist in the field of “Dance Movement Therapy (DMT)” summarizing the effects of this movement-oriented form of psychotherapy on different outcomes (Strassel et al., 2011; Kiepe et al., 2012; Koch et al., 2014). For example, Strassel et al. undertook a systematic review of randomized controlled trials and reviews on the effects of DMT (Strassel et al., 2011). They state that benefits in terms of quality of life, self-esteem, or coping with a disease are the most reported positive effects. However, the identified reviews that examine the impact of dance interventions concentrate on certain populations, study approaches, settings, outcomes or physical benefits in particular (Keogh et al., 2009; Burkhardt and Brennan, 2012; Kiepe et al., 2012; Guzmán-García et al., 2013; Hwang and Braun, 2015; Rodrigues-Krause et al., 2016). Two reviews point out the benefits of art projects without considering the inherent value of dance on its own (Daykin et al., 2008; Bungay and Vella-Burrows, 2013).

Therefore, this *systematic review aims* at giving an overview of studies that investigate the effects of a dance intervention on aspects of the participants' self. We include studies with quantitative as well as qualitative methodologies following the call of making use of all forms of evidence (Dixon-Woods et al., 2005). In detail, this review focuses the following *research questions*: (a) What is the evidence of the reported effects on different aspects of the self in children/adolescents and in adults? (b) Which study approaches and designs are used and what characterizes the interventions (contents, timeframes)? (c) What are the qualitative facets of the implemented studies and what are issues for future research?

## Methods

The systematic review was conducted according to the PRISMA guidelines (Moher et al., 2015). PRISMA provides checklists and recommendations to authors of reviews and meta-analyses to ensure transparency, validity, and reproducibility.

### Search strategy

One author (TS) searched the electronic databases Web of Science, Medline, Psyndex, PsycINFO, PsycARTICLES, Teacher Reference Center, Education Source, ERIC, SPORTDiscus, SocINDEX, and Scopus for relevant studies. Language was restricted to English or German. There were no limitations concerning the publication period. The search included three main aspects: (i) Outcome/topic “aspects of the self”; (ii) Intervention; (iii) Content “dance.” The following search term was used (e.g., Web of Science): (“Self competenc^*^” OR Self OR Perception^*^ OR “Physic^*^ Competenc^*^” OR Abilit^*^ OR Image^*^ OR Awareness^*^ OR Identit^*^ OR Personal^*^) AND (Intervention^*^ OR Program^*^ OR Training^*^ OR Experiment^*^ OR Treatment^*^ OR Participation^*^ OR Instruction^*^ OR Exercise^*^ OR Course^*^) AND (Danc^*^ OR “Hip Hop” OR Ballet). The first data collection was completed in May 2016. An update search was undertaken in March 2017 (Date of last search: 15.03.17). We used the same search term but made an additional restriction to the publication date. The review protocol can be found on “PROSPERO”^1^ under the registration number CRD42016039288. The protocol presents additional information concerning the search levels and the search terms for all databases.

### Inclusion and exclusion criteria

Table 1 contains a list of the terms determining our search and reasons for inclusion/exclusion. We used the following eligibility criteria: (i) The study had to be an intervention study. Any type of intervention study was included (qualitative and quantitative approaches, randomized control trials, quasi-experimental design trials, pilot studies). (ii) The study had to investigate at least one aspect of the self. The terms that arise from the definitions built the base for our search. (iii) The intervention content had to be dance. Studies with any kind of dance forms are included. (iv) The study had to be published as a journal article in English or German. We decided to include only journal articles to guarantee a certain quality standard of our included studies in terms of e.g., content-related, methodical, linguistic, and structural aspects.

**Table 1:** Inclusion and exclusion criteria.

**Inclusion criteria**	**Exclusion criteria**
Intervention study	Combined physical activities programs
Investigation of at least one aspect of the self: (physical) self-concept,	Forms of (step) aerobic dance
physical-/self-competence, (physical) self-perception, self-/body-image,	Dance exergames
self-efficacy, self-esteem, self-perceived abilities	Dance Movement Therapy
Dance as intervention content: hip hop dance, ballet, modern dance,	Programs for vocational dancers or in vocational training
contemporary dance, jazz dance, social dance/ballroom dance (e.g., waltz, jive, latin, tango), country & western dance, folk dance, line dance, square dance, contra dance, step dance	Interventions with only one dance session
English or German language	
Publication as journal article	

Intervention studies were excluded if dance was only one part among other contents (e.g., general physical activities programs), because the inherent value of dance cannot be captured. Moreover, these programs differ in terms of their composition of different sport types/subfields and the number of subfields beside dance. Studies working with dance labeled as “Dance Movement Therapy (DMT)” were also excluded. DMT is an internationally accepted body-oriented form of psychotherapy (not every dance intervention with people with special disorders, e.g., physical disabilities, is likewise a dance movement therapeutic intervention; Schmais and White, 1986; Zitomer and Reid, 2011). We excluded studies including dance workshops for vocational dancers or studies that considered the vocational training itself as an intervention. Dance exergames, e.g., “Dance Dance Revolution (DDR),” were excluded because of the lack of internal orientation concerning movement awareness (next level, break the high-score) and the limitation of movement concerning space, quality and variation of movement patterns due to the frameworks (tapping and stepping on the spot). Moreover, dance provides the opportunity for self-expression and is characterized as an expressive activity. In DDR this inherent characteristic of dance is not of relevance. This also applies for forms of (step) aerobic dance. Furthermore, the main objective of aerobic dance is to train the cardiovascular system and the precise performance of each part of the movement plays a less important role than in dance. Therefore, dance-games as well as aerobic dance forms are less dance but rather an aerobic/fitness and coordination training. The exclusion of forms of aerobic dance is in accordance with the systematic review of Hwang and Braun (2015). The exclusion of studies working with DMT, dance exergames and vocational dancers was performed in the review of Burkhardt and Brennan (2012). Additionally, we excluded one study that offered only one dance session.

### Selection process and risk of bias assessment

In a first step, two reviewers (TS, CO) independently screened all titles of the extracted references. Potentially relevant studies meeting the eligibility criteria were included. Undefined titles remained, due to the risk of bias. The abstracts of included studies were screened using the same procedure. If the abstract information was not sufficient to assess eligibility, references remained for full-text analysis. All included relevant studies were assessed based on their full-text for inclusion or exclusion. Both reviewers carefully documented reasons for exclusion after abstract and full-text screening. Furthermore, one author (TS) screened reference lists and citations of included full-text articles in the databases Scopus, Web of Science and the EBSCOhost sources to identify additional relevant studies. Discrepancies between article selections were resolved after discussion at the end of each step of the selection process. In cases of disagreement we involved a third reviewer (SS). The update search followed the same selection procedure.

### Data extraction

We developed a data extraction sheet and extracted the following data from the full-text articles:

(i) Study characteristics: citation, author, date of publication, country, journal, study approach and design, data acquisition period
(ii) Population: age, gender, sample size, special disorders/impairments/needs (mental, physical)
(iii) Setting
(iv) Intervention characteristics: intervention content, duration and frequency, control group treatment
(v) Methodology and analytic process
(vi) Reported outcomes and main results
(vii) Barriers and limitations
(viii) Information for assessment of the risk of bias

One author (TS) extracted the data of the full-text articles and a second author (CO) checked the data. If data were missing, we contacted the author for further information. Out of six contacted authors (email to the corresponding author), one responded and provided the missing information.

### Analysis and synthesis

Due to the heterogeneity of the included studies, options for statistical quantitative analyses and overall synthesis of the research findings were limited. Therefore, we decided to analyze the study results for children/adolescents and adults separately in a narrative summary combined with a thematic analysis by the reported constructs (Dixon-Woods et al., 2005). Additionally, we provide three tables presenting the framework conditions and main results of each selected study by separating qualitative, quantitative and mixed methods studies (see Tables 2–4). Moreover, Table 5 contains the available details of the intervention content of included studies.

**Table 2:** Framework conditions of the qualitative studies.

**Authors and date Sample size**	**Sample Setting Country**	**Phenomenon of interest**	**Study design**	**Study aim**	**Evaluation in terms of aspects of the self**
**(A) CHILDREN AND ADOLESCENTS**					
Duberg et al., 2016 *N* = 24	Female adolescents with internalizing problems; age: 13–18 years Centrally located gym	African dance, jazz-dance, contemporary dance (Duberg et al., 2013) (Intervention with a duration of 32 weeks and a frequency of 2 days a week, 75 min per session)	Data collection after the intervention (embedded in an RCT, *N* = 112); 24 participants of the experimental group (*N* = 59) were chosen to be interviewed Qpen-ended interviews	Exploration of the experiences of participating in a dance intervention	Analysis resulted in one main category “finding embodied self-trust that opens new doors” and five generic categories (e.g., “finding acceptance and trust in own ability”).
Jounghwa et al., 2013 *N* = 55	Adolescents (44 female, 11 male); age: N/A (10th-grade students) Art high school Korea	Creative dance (Intervention with a duration of 8 weeks and a frequency of 2 days a week, 50 min per session)	Data collection during and after each dance session Students' reflective drawings and written logs, video recordings of program sessions, investigators' field notes	Investigation of the influence of creative dance on Korean high school students' self-expression and the perceptions about learning creative dance	Topics of the analysis: “expanding self-expression” including using the body as an effective vehicle for communication, “enhancing positive perceptions about the body,”
Zitomer and Reid, 2011 *N* = 14	Children with physical disabilities (*N* = 5) and without disabilities (*N* = 9); age: 6–9 years (*M* = 7.6) YMCA venue Canada	Integrated dance program (Intervention with a duration of 10 weeks and a frequency of 1 day a week, 60 min per session)	Data collection after the intervention Focus group interviews, field notes, observations	Investigation of the children's perceptions on dance ability and disability	Pre-program interview: “can't walk, can't dance.” Post-program interview: “can't walk, but can dance” (able-bodied children's perceptions of disabled children's dance ability), “competence” (perceptions of disabled children of their own dance ability).
**(B) ADULTS**					
Thornberg et al., 2012 *N* = 13	Adults (8 female, 5 male); age: 61–89 years No clear information about setting Sweden	Improvisational/ creative dance (Dance project with a duration of 16 weeks and a frequency of 1–2 days a week including dance workshops and performances)	Data collection after the intervention; selection of 17 participants through an audition procedure (13 agreed to be interviewed) Qualitative open interviews and DVD recordings	Development of knowledge of elderly persons' experiences of participating in a dance workshop	Analysis resulted in two topics: “a surprising awareness about the connection between body and mind” (self-/body awareness), “participation leads to personal growth” (self-development).

**Table 3:** Framework conditions of the quantitative studies.

**Authors and date Sample size**	**Sample Setting Country**	**Intervention**		**Study design**	**Outcomes in aspects of the self and measurements**	**Results in aspects of the self**
		**Content Control group (CG) treatment**	**Duration Frequency**			
**(A) CHILDREN AND ADOLESCENTS**						
Blackman et al., 1988 *N* = 16	Female adolescents; age: *M* = 14.10 High school USA	Dance team participation CG: participation in physical education classes; no participation in extracurricular activities	16 weeks Summer-period: 2–3 h 4–5 times a week plus 4-day dance team camp, school period: 1 h daily plus performances	Pre-post-test design for experimental; post-test design for control; no randomization for experimental (*N* = 8) and control (*N* = 8)	Self-concept (Tennessee Self-Concept Scale; Roid and Fitts, 1991) Self-esteem (Self-Esteem Inventory; Coopersmith, 1968) Attitudes toward own body (Body Cathexis Scale; Secord and Jourard, 1953)	Improvement only for physical and social subscales of self-concept in dance group. Lower self-concept mean score for control than for dance group, but no significant differences. No improvement and group-differences for the attitudes toward own body and self-esteem.
Connolly et al., 2011 *N* = 55	Female adolescents; age: 14 years Secondary schools UK	Contemporary dance classes	5–12 h duration depending on school, 60–90 min per session	Pre-post-test design; no control	Self-esteem (Rosenberg Self-Esteem Scale; Rosenberg, 1965)	Improvement in self-esteem.
Jago et al., 2015 *N* = 508	Female adolescents; age: 11–12 years 18 secondary schools (26–33 students per school) UK	After-school dance program with wide range of dance styles CG: only data provision, no treatment	20 weeks 2 days a week, 75 min per session	Cluster randomized controlled trial; pre-post-test (baseline—after 17–20 weeks) and follow-up (after 52 weeks); randomization of the schools to intervention (*N* = 284) or control (*N* = 287) with 9 schools per group; drop-out *N* = 63	Self-esteem (Self-Description Questionnaire; Marsh, 1992)	No improvement of self-esteem in experimental and control group. Higher scores of self-esteem in the control group at T1 and T2 (no differences between groups at T0).
Romero, 2012 *N* = 56	Adolescents (40 female, 32 male); age: 11–16 years Charter middle school (3 health/science classes) USA	Hip hop dance	5 weeks 2 days a week, 50 min per session	Pre-post-test design; recruited *N* = 81; no control; drop-out *N* = 25	Self-efficacy (Questionnaire items based on the California Healthy Kids Survey and the Ambient Hazards Scale; Heath et al., 1993)	Improvement in self-efficacy among girls, not among boys.
Roswal et al., 1988 *N* = 35	Mentally retarded adolescents (12 female, 23 male); age: 11–16 years (*M* = 12.88 for data based group; *M* = 13.47 for creative dance group) Special education classes in three cities USA	Comparison of two dance pedagogical programs (“Data-based dance” vs. creative dance)	8 weeks 5 days a week, 30 min per session	Quasi-experimental comparative study; pre-post-test design; quasi randomization for “data-based dance group” (*N* = 18) or “creative dance group” (*N* = 17) (classes split half and half); no control	Self-concept (Self-Concept Scale; Martinek and Zaichkowsky, 1977)	No improvement of self-concept in both groups.
Studer-Lüthi and Züger, 2012 *N* = 51	Children (28 female, 23 male); age: *M* = 11.3 for experimental, *M* = 11.2 for control Primary school Switzerland	Creative dance CG: non-contact group, school's regular program	4 weeks 2 days a week, 45 min per session	Quasi-experimental study; pre-post-test design; quasi-randomization for experimental (N = 16) and control (N = 35); 1 class for experimental, 2 for control	Body Self-concept (Frankfurter Körperkonzeptskala; Deusinger, 1998)	Improvement of body self-concept (group effect: partial η2 = 0.10)—self-acceptance in particular—in experimental group. No improvement in control group.
**(B) ADULTS**						
Aşçi, 2002 *N* = 138	Young adults (73 female, 65 male); age: 18–27 years University, elective courses at the Physical Education and Sport Department Turkey	Step dance CG: no participation in any regular PA	10 weeks 3 days a week, 50 min per session	Randomized controlled trial; pre-post-test design; randomization for experimental (*N* = 80) and control (*N* = 80); drop-out *N* = 22	Physical self-perception (Physical Self-Perception Profile; Fox and Corbin, 1989)	Improvement of physical self-perception. Participants in the experimental group improved more than participants in the control group (effect of time: η2 = 0.17, group × time interaction effect: η2 = 0.10).
Aşçi, 2009 *N* = 138	Young adults (73 female, 65 male); age: 18–27 years University, elective courses at the Physical Education and Sport Department Turkey	Step dance CG: no participation in any organized or structured exercise, participation in a lecture about physiological and psychological benefits of exercise	10 weeks 3 days a week, 50 min per session	Randomized controlled trial; pre-post-test design; randomization for experimental (*N* = 80) and control (*N* = 80); drop-out *N* = 22	Self-concept (Tennessee Self-Concept Scale; Roid and Fitts, 1991)	Improvement only for physical self (effect of time: η2 = 0.05, treatment × time interaction effect: η2 = 0.06) and identity dimensions (treatment × time interaction effect: η2 = 0.03) of self-concept for the experimental group compared to control.
Baptista et al., 2012 *N* = 80	Female adults with fibromyalgia not having altered treatment in previous four weeks; age: 18–65 years No clear information about setting Brazil	Belly dance CG: remaining on a waiting list	16 weeks 2 days a week, 60 min per session	Randomized controlled trial; pre-post-test (baseline - after 16 weeks) and follow-up (after 32 weeks); randomization for experimental (*N* = 40) and control (*N* = 40); drop-out *N* = 5 (data-repetition)	Self-image (Body Dysmorphic Disorder Examination; Jorge et al., 2008)	Improvement for self-image in dance group while control group remained stable. Improvements in the dance group between TO/T1 as well as between T1/T2.
Fonseca et al., 2014 *N* = 30	Adults; age: 21–60 years (*M* = 35.9 for experimental; *M* = 38.9 for control) School of ballroom dancing Brazil	Ballroom dancing CG: no participation in any PA, but four lectures on body perception (2 hours per lecture)	12 weeks 1 day a week, 90 min per session	Pre-post-test design; no information about a conducted randomization for experimental (*N* = 15) and control (*N* = 15)	Body size perception/body schema (Adaption of the “Image Marking Procedure”; Askevold, 1975)	No group differences associated with ballroom dance. Intragroup pre-post comparison show positive tendencies for dance group: dance group shows an increase of 40% while control group shows a 22% decrease in the number of participants who have appropriate body perception.
Kosmat and Vranic, 2017 *N* = 22	Adults (15 female, 9 male); age: 69–88 years (*M* = 80.8) Residential care center Croatia	Dance intervention (inter alia slow waltz) CG: alternative active non-dance program	10 weeks 1 day a week, 45 min per session	Randomized controlled trial; pre-post-test and follow-up (after 5 months); randomization for experimental (*N* = 12) and control (*N* = 12); drop-out *N* = 2	Self-efficacy (General Self-Efficacy Scale; Schwarzer and Jerusalem, 1995)	No improvement in self-efficacy and no group differences.
Mandelbaum et al., 2016 *N* = 8	Adults with multiple sclerosis (“independent community ambulators”); age: 29–63 years No clear information about setting USA	Salsa dance	4 weeks 2 days a week, 60 min per session	Pilot study; pre-post-test and follow-ups (after 3 and 6 months); no control	Self-efficacy (Multiple Sclerosis Self-Efficacy Scale; Rigby et al., 2003)	No improvement in self-efficacy.
Pinniger et al., 2012 *N* = 66	Adults with self-reported stress, anxiety, and/or depression; age: 18–80 years (*M* = 44.39) Community/art venue Australia	Argentine tango dance CG: mindfulness meditation classes or waiting-list control group	6 weeks 1 day a week, 90 min per session	Randomized controlled trial; pre-post-test design; randomization for experimental (*N* = 33) and controls (meditation group: *N* = 33, waiting-control: *N* = 31); drop-out *N* = 31	Self-esteem (Rosenberg Self Esteem Scale; Rosenberg, 1965)	No improvement in self-esteem for all groups.
Pinniger et al., 2013a *N* = 17	Women with age-related macular degeneration; age: 65 years and older Community center Australia	Argentine tango dance CG: waiting-list control	4 weeks 2 days a week, 90 min per session	Feasibility Study; randomized controlled trial; pre-post-test design; randomization for experimental (*N* = 8) and waiting-control (*N* = 9)	Self-esteem (Rosenberg Self Esteem Scale; Rosenberg, 1965)	Improvement of dance participants in self-esteem relative to controls (group effect: partial η2 = .89).
Pinniger et al., 2013b *N* = 64	Adults with self-reported affective symptoms; age: 18–68 years (*M* = 39.5) Community/art venue Australia	Argentine tango dance CG: waiting-list control	8 weeks 1 day a week, 90 min per session	Randomized controlled trial; pre-post-test (baseline -after 8 weeks) and follow-up (after 1 month); randomization for experimentals (dance group: *N* = 24, meditation group: *N* = 24, exercise group: *N* = 24) and waiting-control (*N* = 25); drop-out *N* = 33	Self-efficacy (General Self-Efficacy Scale; Schwarzer and Jerusalem, 1995)	No improvement in self-efficacy for dance and meditation groups. Improvement in self-efficacy for exercise group at post-test and follow-up. Waiting-control group maintained similar scores.
Pinniger et al., 2013c *N* = 41	Adults with self-reported affective symptoms; age: 18–73 years (*M* = 38.68) Community/art venue Australia	Intensive tango dance CG: waiting-list control group	2 weeks 4 days a week, 90 min per session	Randomized controlled trial; pre-post-test (baseline - after 2 weeks) and follow-up (after 1 month); randomization for experimental (*N* = 20) and waiting-control (*N* = 24); drop-out *N* = 3	Self-efficacy (General Self-Efficacy Scale; Schwarzer and Jerusalem, 1995)	Improvement in self-efficacy for tango group at post-test relative to control (group effect: partial η2 = 0.19). No differences between pre-test and follow-up for self-efficacy.
Soares Costa de Mendonça et al., 2015 *N* = 89	Female adults; age: 25–55 years (*M* = 41.42) Specific locations for groups (gyms, public municipal institution) Brazil	Brazilian folk dance CG: sedentary control group with subjects reporting no regular PA-exercise during the previous 6 months. Subjects were asked not to start any exercise program but maintain their regular activities of daily life.	16 weeks 3 days a week, 50–60 min per session	Cluster-randomization for experimental groups (dance group: *N* = 18, strength training group: *N* = 25, hydrogymnastics group: *N* = 21) and control (*N* = 25)	Satisfaction with physical appearance, SPA (self-designed question) Body image perception, BIP (Stunkard Scale of Silhouettes for Adults; Stunkard et al., 1983) Self-esteem, SE, (Rosenberg Self-Esteem Scale; Rosenberg, 1965)	Improvements for all experimental groups in SPA (time effect: η2 = 0.56, time × group interaction effect: η2 = 0.23), SE (time effect: η2 = 0.11, group effect: η2 = 0.15). No effect for BIP, but less dissatisfaction for subjects in experimental than control. No effects for control-subjects.

**Table 4:** Framework conditions of the mixed methods studies.

**Authors and date Sample size**	**Sample Setting Country**	**Intervention**		**Study design**	**Outcomes in aspects of the self and quantitative measurements Study aim and qualitative methods**	**Results in aspects of the self**
		**Content Control group (CG) treatment**	**Duration Frequency**			
**(A) CHILDREN AND ADOLESCENTS**						
Backe and Graefe, 2004 *N* = 9	Female children with lack of self-esteem; age: 8–10 years University, movement outpatient clinic (“Bewegungs-ambulatorium”) Germany	Educational dance/creative dance	12 weeks 1 day a week, 90 min per session, final presentation	Pilot study; empirical experimental method & qualitative phenomenological approach; pre-post-test design; no control	Self-esteem (Aussageliste zum Selbstwertgefühl; Schauder, 1991) Investigation of an interaction between self-esteem and movement behavior and general behavior by observations	Improvement of global self-esteem in 7 of 9 girls, but no calculation of significance. Parents and teachers rated self-esteem higher at post-test compared to pre-test and the girls' self-evaluations. Support of the hypothesis of a correlation between movement behavior change and increase of self-esteem by qualitative observations.
Caf et al., 1997 *N* = 16	Hypoactive children with learning difficulties (10 female, 6 male); age: 7–10 years Primary school Slovenia	Creative movement and dance CG: no participation in any optional activity outside of the ordinary classroom schoolwork	16 weeks 1 day a week, 60 min per session	Pilot study; empirical experimental method & action research approach; pre-post-test design; no randomization for experimental (N = 8) and control (N = 8)	Body image (Body Image Evaluating Scale; Cratty, 1979) Investigation of the children's behavior activities and qualities in different categories, e.g., body image, by observations and teacher diary notes	Improvement in the tasks “body sides” and “objects” for experimental group in body image. No presentation of results in terms of body image from the qualitative observations.
**(B) ADULTS**						
Stickley et al., 2015 *N* = 330	Adults (of 34 dance classes); age: wide age range, no precise information Rural community venue UK	Community-based dance activities with a range of dance styles	30 months (attendance of at least 8 individual classes for inclusion in analysis)	Post-test design: questionnaire after 8–10 weeks (providing questionnaire *N* = 602, response rate 55%); focus group discussions with 13 participants immediately after dance sessions; interviews among 7 participants; no control	Attitudes about health and well-being with a question about self-expression (Stickley et al., 2015) Investigation of reasons for participating and the gained benefits of participating in dance sessions by focus group discussions and individual interviews	57% agreed with the statement of an increased ability to express themselves (42.1% neutral; 1% disagree). Key topics of the focus group discussions and individual interviews: development of a feeling “like you've achieved something” (self-efficacy) and a “gain [of] confidence” (self-confidence), “expressing yourself” (self-expression).

**Table 5:** Details of the intervention content of included studies.

**Source**	**Dance content/style**	**Basic theory/methodical-didactical concepts**	**Teacher**	**Participation type**
Aşçi, 2002	Step dance	Sessions composed of warm-up, step dance, floor exercises, cool-down; intensity: 60–80% of heart rate reserves	No information	Voluntary
Aşçi, 2009	Step dance	Sessions composed of warm-up, step dance, floor exercises, cool-down; intensity: 60–80% of heart rate reserves	No information	Voluntary
Backe and Graefe, 2004	Educational dance/creative dance (Modern dance, Jazz-dance, New dance)^*^	Program based on Laban (1988), Haselbach (1976) and Neuber (2000); deductive as well as inductive methods; emphasis on movement-improvisation/-exploration and creation as methods to promote self-esteem; exploration of the own character using the “four elements”; focus on individualization; positive feedback-method; final presentation	No clear information (researchers?)	Voluntary
Baptista et al., 2012	Belly dance	Sessions composed of warm-up, predetermined movements of the day, choreography, cool-down; additional home training	Experienced physiotherapist	No clear information about participation type
Blackman et al., 1988	Dance team participation	Practice consisting of learning and perfecting dance routines (combinations of high kicks, jumping, vigorous arm, leg, trunk, and hip movements); individual development of new routines; performances at football games	No information	Voluntary
Caf et al., 1997	Creative dance	Improvization, exploration and creation of movements	No clear information	Voluntary
Connolly et al., 2011	Contemporary dance	Dynamic classes with emphasis on building muscular strength; technique exercises (half of each lesson) as well as creative/choreographic tasks; focus on autonomy of choice, progression and development of movement phrases, peer critique and encouragement, self-improvement and motivation, enjoyable learning environment; program planned by dance artists	Local dance artists	Voluntary or obligatory participation (depending on school)
Fonseca et al., 2014	Ballroom dancing	Non-competitive program for beginners; teaching of technical content (posture, leading, rhythmic perception, step performance) and sequences of predetermined movements; dance partners alternated frequently; intensity: mild to moderate	Dance school teachers	Voluntary
Duberg et al., 2016	African dance, Jazz-dance, Contemporary dance	Program based on Self-Determination Theory (Ryan and Deci, 2000); main focus on enjoyment of movement; mainly deductive method with space for some improvisation; promotion of body-awareness; participants provide input regarding music and dance styles; no performances	Trained dance instructors	Voluntary
Jago et al., 2015	No information about specific dance style/s (wide range of styles)	After-school dance program; standardised programme: focus on building participants' perceived autonomy to be active and perceived dance competence in a social, autonomy-supportive environment	External, trained dance instructors	Voluntary
Jounghwa et al., 2013	Creative dance	Program based on Laban (1988) and Jeon (2005); exploration and creation of movements as main content, individual and group activities, final presentation	First investigator and cooperating teacher	Obligatory (part of curriculum)
Kosmat and Vranic, 2017	No clear information about dance style/s (slow-waltz amongst others?)	Dance intervention developed for older adults by a trained dance pedagogue; sessions composed of warm-up, deductive method (learning of a choreography), dancing slow-waltz, self-practice and feedback	Trained dancing master (experimenter)	Voluntary
Mandelbaum et al., 2016	Salsa dance	Sessions composed of learning and practice of dance steps, frame-posture-balance, leader/follower roles, stretching for people with multiple sclerosis; dance partners alternated frequently; additional self-practice of 30 min/week at home to increase physical activity	Professional dance instructor with experience in teaching people with disabilities	Voluntary
Pinniger et al., 2012	Argentine tango dance	Lessons based on the Argentinean Close-embrace Tradition; sessions focus on different aspects of the dance (e.g., consciousness of walking, awareness of one's own and partner's body, resistance and transference of weight, close embrace); emphasis on the enjoyment rather than achieving a particular outcome	Experienced, registered instructor	Voluntary
Pinniger et al., 2013a	Argentine tango dance	Program adapted for people with macular degeneration; trained tango-helpers; learning and practice of correct posture, transference of weight and tango steps	Experienced tango instructor	Voluntary
Pinniger et al., 2013b	Argentine tango dance	Lessons based on the Argentinean Close-embrace Tradition; sessions focus on different aspects of the dance (e.g., consciousness of walking, awareness of one's own and partner's body, resistance and transference of weight, close embrace); emphasis on the enjoyment rather than achieving a particular outcome	Experienced, registered instructor	Voluntary
Pinniger et al., 2013c	Argentine tango dance	Intensive program designed to encourage rapid learning and to minimize anxieties; experienced tango-helpers; sessions focus on different aspects of the dance (e.g., consciousness of walking, awareness of one's own and partner's body, resistance and transference of weight); individualization of dance challenges	Instructor with effective interpersonal skills	Voluntary
Romero, 2012	Hip Hop dance	Intervention key-components based on Social-Cognitive Theory and the Critical Hip Hop Pedagogy (Akom, 2009); program composed of interactive sessions on lesson contents (e.g., Hip Hop culture), break-dancing, individual practice, one-on-one assistance, group battles	No clear information (“culturally similar social role models”?)	Voluntary
Roswal et al., 1988	Comparison of two dance pedagogical programs	“Data-based dance” program (Dunn et al., 1979) vs. creative dance program (Fitt and Riordan, 1980); two contrary pedagogies concerning student participation (individual vs. simultaneously as group), objective achievement (direct vs. indirect), movement solutions (one vs. various correct solutions), positive reinforcement (task-specific vs. general to participation), learning (stimulus-response vs. exploration or problem solving), success (accomplishment of specific tasks vs. value of moving and discovering)	Trained special education teachers	Obligatory
Soares Costa de Mendonça et al., 2015	Brazilian folk dance	Program as aerobic training; rhythmic variations of Brazilian folk dance; intensity: 60–85% of heart rate exercises	No information	Voluntary
Stickley et al., 2015	No information about specific dance style/s (wide range of styles)	Community-based dance activities; program aims to raise awareness of the health and well-being benefits of dance and to support the development of locally based dance classes	Trained volunteers and professional dance artists	Voluntary
Studer-Lüthi and Züger, 2012	Creative dance	Program based on Laban (1981) and Kestenberg et al. (1999); sessions composed of deductive methods as well as movement-exploration and creation, group works, tasks to promote body-awareness, meditation	Experienced dance therapist	Obligatory
Thornberg et al., 2012	Improvisational/ creative dance	Dance workshop together with professional dancers; improvisation of movements that express different emotions and give form to a personal memory; choreographer created dance piece out of these memories; seven public performances	Professional choreographer	Voluntary
Zitomer and Reid, 2011	Integrated dance program	Based on the principles of Contact Theory (Allport, 1954): equal status, common goals, cooperation/group works and authority support between children with and without disabilities; focus on exploration of different executive modes of movement sequences	Researcher and assistants (all able-bodied)	Voluntary

* *The term “creative dance” describes a methodical approach to teach dance. Nevertheless, some authors use the term as a substitute for the dance content and/or style. Backe & Graefe present both dance styles and methodical focus*.

### Methodological quality assessment

Two reviewers (TS, SS) assessed the methodological quality independently. Initial inter-rater reliability on all assessed studies was moderate for studies with quantitative methodologies (χ = 0.77) and strong for studies with qualitative methodologies (χ = 0.84) (McHugh, 2012). Discrepancies were solved through discussion or by referring to a third reviewer (CO). For assessing the quality of studies with qualitative measures we used the “Checklist for Qualitative Research” provided by the Joanna Briggs Institute (JBI) (Lockwood et al., 2015). To assess the quality of studies with quantitative methodologies we used the “Quantitative Research Assessment Tool” provided by the Child Care and Early Education Research Connections (CCEERC)^2^ Most of the assessment tools include criteria that can hardly be fulfilled in a teaching context, e.g., “Performance Bias”: Blinding of participants and personnel^3^ For that reason, we chose a tool with an educational background (as many of the identified studies took place in an educational setting). Quantitative studies were rated on 11 questions using the following scale: 1, 0, −1, not applicable. A question concerning the ethical approval was added in the quantitative assessment tool to account for completeness (Q 12). The adapted tool is part of the Supplementary Material. Additionally, we calculated the mean of the ratings for each quantitative study. Qualitative studies were rated on 10 questions using the following scale: yes, no, unclear, not applicable. The results of the appraisal for each study are presented in Table 6 for qualitative studies and in Table 7 for quantitative studies. Table 7 functions as a summary table, which highlights important quality criteria like sample size, effect size measures, randomization and control. A table with the exhaustive results of the quality assessment in quantitative studies is provided in the supplementary files (see Table A1 in Supplementary Material). The tables should be considered in interpreting the study findings. We did not exclude studies based on poor methodological quality. The respective studies provide important information for further research concerning research desiderata.

**Table 6:** Quality of studies with qualitative methodologies (4 qualitative, 3 mixed).

**Source**	**Q1**	**Q2**	**Q3**	**Q4**	**Q5**	**Q6**	**Q7**	**Q8**	**Q9**	**Q10**
**(A) CHILDREN AND ADOLESCENTS**										
Backe and Graefe, 2004	No	No	Yes	No	No	Yes	No	No	No	No
Caf et al., 1997	No	Unclear	Yes	No	Unclear	Yes	Unclear	No	No	No
Duberg et al., 2016	Yes	Yes	Yes	Yes	Yes	Yes	Yes	Yes	Yes	Yes
Jounghwa et al., 2013	Yes	Yes	Yes	Yes	Yes	No	No	Yes	No	Yes
Zitomer and Reid, 2011	Yes	Yes	Yes	Yes	Yes	Yes	Unclear	Yes	Yes	Yes
**(B) ADULTS**										
Stickley et al., 2015	No	No	Yes	Yes	No	No	No	Yes	Yes	Yes
Thornberg et al., 2012	Yes	Yes	Yes	Yes	Yes	No	Unclear	Yes	Yes	Yes

Q1: Is there congruity between the stated philosophical perspective and the research methodology?

Q2: Is there congruity between the research methodology and the research question or objectives?

Q3: Is there congruity between the research methodology and the methods used to collect data?

Q4: Is there congruity between the research methodology and the representation and analysis of data?

Q5: Is there congruity between the research methodology and the interpretation of results?

Q6: Is there a statement locating the researcher culturally or theoretically?

Q7: Is the influence of the researcher on the research, and vice-versa, addressed?

Q8: Are the participants, and their voices, adequately represented?

Q9: Is the research ethical according to current criteria or, for recent studies, and is there evidence of ethical approval by an appropriate body?

Q10: Do the conclusions drawn in the research report flow from the analysis, or interpretation, of the data?

**Table 7:** Summary of the studies' quality with quantitative methodologies (17 quantitative, 3 mixed).

**Source**	**Sample size**	**Control group?**	**Randomization?**	**Effect size measures?**	**Overall quality**
**(A) CHILDREN AND ADOLESCENTS**					
Backe and Graefe, 2004	9	No	No	No	↓
Baptista et al., 2012	80	Yes	Yes	No	↑
Blackman et al., 1988	16	Yes, but no pre-test for control	No	No	→
Caf et al., 1997	16	Yes	No	No	↓
Connolly et al., 2011	55	No	No	No	→
Jago et al., 2015	508	Yes	Yes	No	↑
Romero, 2012	56	No	No	No	→
Roswal et al., 1988	35	No	Yes (for two IGs)	No	↓
Studer-Lüthi and Züger, 2012	51	Yes	Yes	Yes	→
**(B) ADULTS**					
Aşçi, 2002	138	Yes	Yes	Yes	↑
Aşçi, 2009	138	Yes	Yes	Yes	↑
Fonseca et al., 2014	30	Yes	No information	Yes	→
Kosmat and Vranic, 2017	22	Yes	Yes	Yes	↑
Mandelbaum et al., 2016	8	No	No	No	→
Pinniger et al., 2012	66	Yes	Yes	Yes	↑
Pinniger et al., 2013a	17	Yes	Yes	Yes	→
Pinniger et al., 2013b	64	Yes	Yes	Yes	↑
Pinniger et al., 2013c	41	Yes	Yes	Yes	↑
Soares Costa de Mendonça et al., 2015	89	Yes	Yes	Yes	→
Stickley et al., 2015	330	No	No	No	↓

*IG, Intervention group; ↑, Mean in the upper third of the quality rating scale (0.33 < mean < 1); →, Mean in the middle third of the rating scale (−0.33 < mean ≤ 0.33); ↓, Mean in the lower third of the rating scale (− 1 < mean ≤ − 0.33)*.

## Results

**Figure 1:**
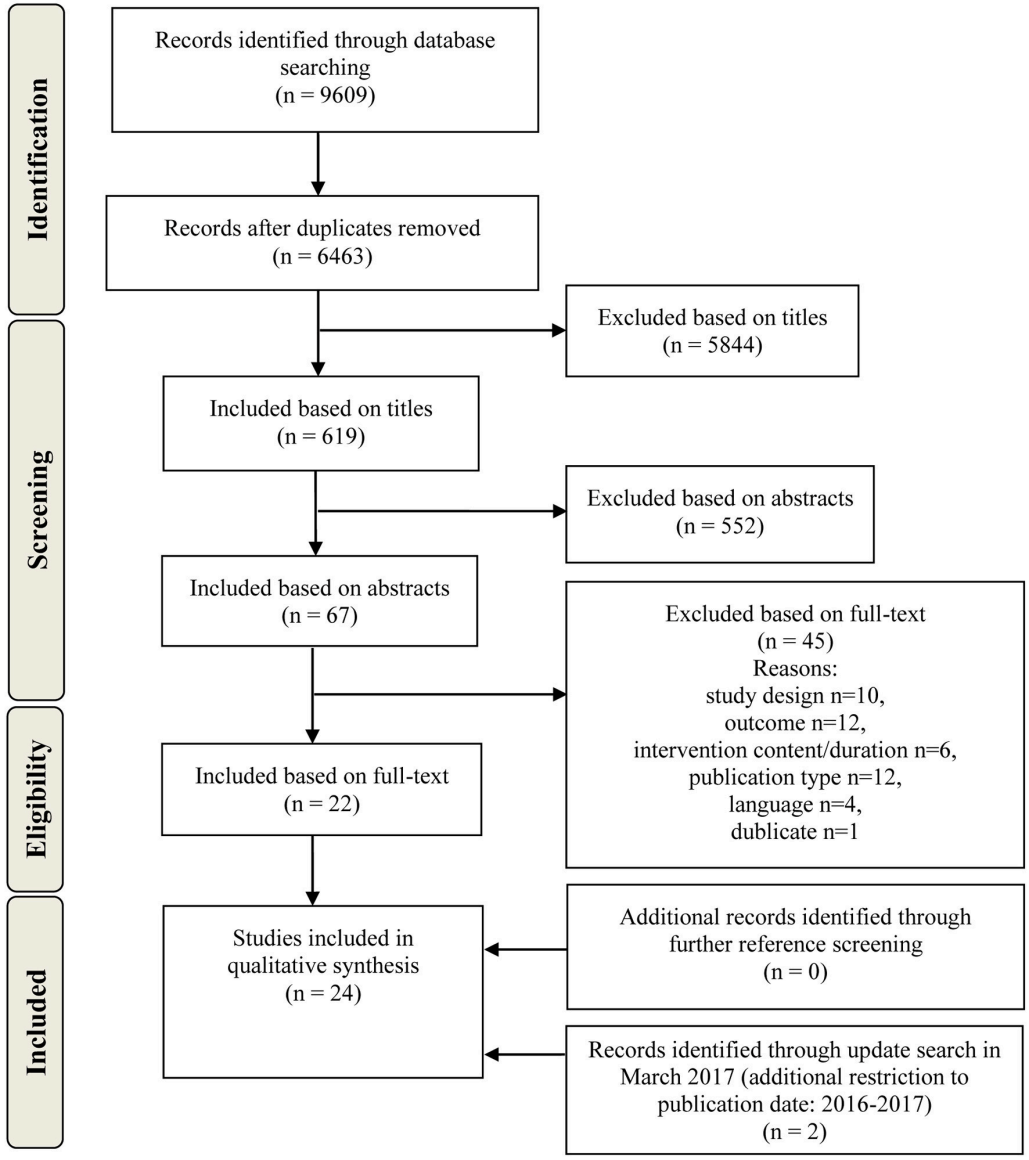
Flow chart of the search and study selection process.

Figure 1 shows the number of extracted references and exclusion/inclusion numbers for each stage of the screening and selection process. Reasons for exclusion after reading the full papers are listed.

The search yielded 9,609 initial hits. After removing duplicates and after screening titles and abstracts, we retrieved 67 articles in full-text. Twenty-two studies met all criteria. Further reference screening yielded no additional studies that met all inclusion criteria. An update search in March 2017 yielded two additional relevant studies. In the end, 24 studies were included in the analysis.

Ten out of 17 quantitative studies report positive effects on various aspects of the self: body-related perceptions (5 out of 8) (Blackman et al., 1988; Aşçi, 2002, 2009; Studer-Lüthi and Züger, 2012; Soares Costa de Mendonça et al., 2015), self-esteem (3 out of 6) (Connolly et al., 2011; Pinniger et al., 2013a; Soares Costa de Mendonça et al., 2015), self-efficacy (2 out of 5) (Romero, 2012; Pinniger et al., 2013c), self-concept/self-image (1 out of 4) (Baptista et al., 2012). All four qualitative studies point out positive development in different aspects of the self as one topic of their analyses: body-related perceptions (Jounghwa et al., 2013), self-trust (Duberg et al., 2016), self-expression (Jounghwa et al., 2013), perception of dance-abilities (Zitomer and Reid, 2011), self-development (Thornberg et al., 2012), self-/body-awareness (Thornberg et al., 2012). All three mixed methods studies report positive tendencies in terms of aspects of the self either in their quantitative results or in both qualitative and quantitative results: body image (Caf et al., 1997), self-esteem (Backe and Graefe, 2004), self-efficacy (Stickley et al., 2015), self-expression (Stickley et al., 2015), self-confidence (Stickley et al., 2015). The studies investigate different populations: Eleven studies investigate children and adolescents (Blackman et al., 1988; Roswal et al., 1988; Caf et al., 1997; Backe and Graefe, 2004; Connolly et al., 2011; Zitomer and Reid, 2011; Romero, 2012; Studer-Lüthi and Züger, 2012; Jounghwa et al., 2013; Jago et al., 2015; Duberg et al., 2016). Thirteen studies examine an adult sample (Aşçi, 2002, 2009; Baptista et al., 2012; Pinniger et al., 2012, 2013a,b,c; Thornberg et al., 2012; Fonseca et al., 2014; Soares Costa de Mendonça et al., 2015; Stickley et al., 2015; Mandelbaum et al., 2016; Kosmat and Vranic, 2017). Eleven studies investigate a population with physical or mental disorders (Roswal et al., 1988; Caf et al., 1997; Backe and Graefe, 2004; Zitomer and Reid, 2011; Baptista et al., 2012; Pinniger et al., 2012, 2013a,b,c; Duberg et al., 2016; Mandelbaum et al., 2016). Over half of the studies are conducted in Europe and North America. Eighteen out of 24 studies were published later than 2010. Overall, the included studies are characterized by a variety of intervention contents and timeframes, research designs/approaches and outcome measures. Due to the studies' heterogeneity and the assumption that specific interventions may have different effects on samples of different age groups the results for children/adolescents and adults are analyzed separately.

### Dance interventions in children and adolescents

#### Population

Seven studies investigate a population of adolescents (age-range 11–18 years) (Blackman et al., 1988; Roswal et al., 1988; Connolly et al., 2011; Romero, 2012; Jounghwa et al., 2013; Jago et al., 2015; Duberg et al., 2016). Four studies investigate a sample of children (age-range 6–11 years) (Caf et al., 1997; Backe and Graefe, 2004; Zitomer and Reid, 2011; Studer-Lüthi and Züger, 2012). Five out of the eleven studies examine a population with special disorders, e.g., mental retardation, physical disabilities (Roswal et al., 1988; Caf et al., 1997; Backe and Graefe, 2004; Zitomer and Reid, 2011; Duberg et al., 2016). Five studies have an all-female sample (Blackman et al., 1988; Backe and Graefe, 2004; Connolly et al., 2011; Jago et al., 2015; Duberg et al., 2016). More than half of the studies examine a sample size of 9–35 participants (Blackman et al., 1988; Roswal et al., 1988; Caf et al., 1997; Backe and Graefe, 2004; Zitomer and Reid, 2011; Duberg et al., 2016). Four studies investigate a sample of 51 to 72 participants (Connolly et al., 2011; Romero, 2012; Studer-Lüthi and Züger, 2012; Jounghwa et al., 2013). One study examines a sample of 508 female adolescents (Jago et al., 2015).

#### Setting

The majority of the studies with a sample of children and adolescents are located in school settings. Five of the eight studies in a school setting take place at secondary schools (Blackman et al., 1988; Connolly et al., 2011; Romero, 2012; Jounghwa et al., 2013; Jago et al., 2015), two at primary schools (Caf et al., 1997; Studer-Lüthi and Züger, 2012) and one intervention is conducted in special education classes (Roswal et al., 1988). Two further studies examining children with special disorders take place at a community venue (Zitomer and Reid, 2011) or at a university (Backe and Graefe, 2004). Another intervention is located at a local gym, but the study does not provide information about the initiator (Duberg et al., 2016).

#### Intervention content and timeframe

The interventions choose different dance styles and contents respectively. Five studies investigate the effects of creative dance programs, including the two studies in primary schools (Roswal et al., 1988; Caf et al., 1997; Backe and Graefe, 2004; Studer-Lüthi and Züger, 2012; Jounghwa et al., 2013). One study compares a creative dance program to a “Data-based dance program” (Roswal et al., 1988). Other intervention contents are hip hop dance (Romero, 2012), contemporary dance (Connolly et al., 2011), an integrated dance program (Zitomer and Reid, 2011) and the participation in a school dance team (Blackman et al., 1988). One study offers various dance styles (African dance, jazz-dance, contemporary dance) (Duberg et al., 2016). One study does not specify the intervention content (Jago et al., 2015). The intervention duration ranges from four (Studer-Lüthi and Züger, 2012) to 32 weeks (Duberg et al., 2016). Frequency ranges from one session a week to daily dance training for the participants in a dance team intervention (Blackman et al., 1988). In seven out of 11 studies, participation is purely voluntary (Blackman et al., 1988; Caf et al., 1997; Backe and Graefe, 2004; Zitomer and Reid, 2011; Romero, 2012; Jago et al., 2015; Duberg et al., 2016).

#### Research approach and design

The studies use different research approaches and designs. Two studies are quasi or rather cluster randomized controlled trials with pre- and post-testing (Studer-Lüthi and Züger, 2012; Jago et al., 2015). One of them includes follow-up measures (Jago et al., 2015). Two studies are non-randomized controlled trials, but with different research approaches [quantitative (Blackman et al., 1988), mixed methods (Caf et al., 1997)] and measurement timeframes, as one study lacks a pretest of the control group (Blackman et al., 1988). Furthermore, there exists one study with a quasi-experimental comparative design without having a non-dance control group (Roswal et al., 1988). Control groups are absent in two additional studies with quantitative methodologies (pre-post-test) (Connolly et al., 2011; Romero, 2012). A mixed methods pilot study lacks a control group, too (Backe and Graefe, 2004). Three studies follow a purely qualitative approach (Zitomer and Reid, 2011; Jounghwa et al., 2013; Duberg et al., 2016). They work with interviews, field notes, observations, video recordings, reflective drawings, and written logs.

#### Investigated aspects of the self and results

Different aspects of the self are identified as main topics in studies with qualitative methodologies. The participants report positive changes in terms of self-trust in their abilities (Duberg et al., 2016) and in terms of the perceptions of dance ability (Zitomer and Reid, 2011). Moreover, one study highlights an enhancement in self-expression and positive perceptions about the body (Jounghwa et al., 2013).

Studies with quantitative methodologies investigate different aspects of the self and yield different effects. Two studies investigate outcomes that can be summarized as “body-related perceptions” and both report positive effects on body self-concept (Blackman et al., 1988; Studer-Lüthi and Züger, 2012). However, one of them does not find positive effects on attitudes toward one's own body (Blackman et al., 1988). Three studies investigate the effects of dance on self-esteem with inconsistent findings. One study reports positive effects (Connolly et al., 2011). Two studies do not find a positive effect (Blackman et al., 1988) or report negative development of self-esteem in the experimental and control group (Jago et al., 2015). Two studies analyze the effects of dance interventions on self-concept (Blackman et al., 1988; Roswal et al., 1988). Both miss out positive effects of the overall construct, but one of them reports an improvement of the physical and social subscale of self-concept (Blackman et al., 1988). Another study confirms a positive effect of hip hop dance on self-efficacy only for girls, not for boys (Romero, 2012).

Two studies chose a mixed methods approach. One study presents positive changes of the children's movement behavior (expansion in the use of space and the dynamic nature of movement) in the qualitative observations (Backe and Graefe, 2004). Positive tendencies in the quantitative measures in terms of self-esteem are associated with the qualitative findings as the progress in movement behavior is seen as an expression of an increased self-esteem (Backe and Graefe, 2004). Nevertheless, the authors do not calculate significance levels. Another study reports positive effects on subscales of body image but does not present results of the qualitative analysis in terms of aspects of the self (Caf et al., 1997).

#### Study quality

The Tables 6, 7 inform about the fulfillment of important quality criteria. The detailed ratings for each quantitative study are presented in Table A1 in Supplementary Material.

Five studies work with qualitative or mixed methods approaches. Three of them (Zitomer and Reid, 2011; Jounghwa et al., 2013; Duberg et al., 2016) meet at least two thirds of the criteria of the methodological assessment tool for qualitative studies. The study conducted by Duberg et al. fulfills all criteria (Duberg et al., 2016). Two studies meet two out of ten criteria (Caf et al., 1997; Backe and Graefe, 2004). All studies show congruity between the research methodology and the methods used to collect data. In contrast, most of the studies fail to address clearly the influence of the researcher on the research, and vice versa. Additionally, three out of five studies do not provide information about ethical considerations (Caf et al., 1997; Backe and Graefe, 2004; Jounghwa et al., 2013).

Eight studies work with quantitative measures. The methodological quality for most of the studies (Blackman et al., 1988; Roswal et al., 1988; Caf et al., 1997; Backe and Graefe, 2004; Connolly et al., 2011; Romero, 2012; Studer-Lüthi and Züger, 2012) ranges in the lower and middle third of the quality assessment scale, with mean values ranging from −0.5 to 0.17. One study is in the upper third with a mean value of 0.58 (Jago et al., 2015). Major reasons for low ratings are small samples, missing coefficients or standard errors for the main effect variables as well as missing information about response-/attrition rates and the handling of missing data. Only one study provides results of effect sizes measures. Five out of eight studies present means and standard deviations/errors for all numeric variables. Two studies select the participants randomly. Half of the studies present statements according to ethical approval in an appropriate way. Most of the studies describe their main variables and concepts of interest and fulfill the operationalization of these concepts.

### Dance interventions in adults

#### Population

Thirteen studies investigate the effects of dance in an adult population. The studies are based on twelve data sets. A wide age range characterizes more than 50 percent of the samples. However, three studies investigate a sample of older adults (Thornberg et al., 2012; Pinniger et al., 2013a; Kosmat and Vranic, 2017). Contrary, Aşçi's studies examine a sample of young adults with an age range of 18–27 years (Aşçi, 2002, 2009). Six studies investigate a population with special disorders (Baptista et al., 2012; Pinniger et al., 2012, 2013a,b,c; Mandelbaum et al., 2016). Six studies investigate a sample ranging from 8 to 41 participants (Thornberg et al., 2012; Pinniger et al., 2013a,c; Fonseca et al., 2014; Mandelbaum et al., 2016; Kosmat and Vranic, 2017). Four studies show a sample size of 64 up to 89 participants (Baptista et al., 2012; Pinniger et al., 2012, 2013b; Soares Costa de Mendonça et al., 2015). Moreover, Aşçi's studies investigate 138 participants (Aşçi, 2002, 2009). Stickley et al. conduct a study with 330 participants consisting of people from 34 different dance classes (Stickley et al., 2015).

#### Setting

The studies are located in different settings. Six studies take place at community/public or art venues (Pinniger et al., 2012, 2013a,b,c; Soares Costa de Mendonça et al., 2015; Stickley et al., 2015) and one study is located in a school for ballroom dancing (Baptista et al., 2012). Another study is conducted in a residential care center (Kosmat and Vranic, 2017). Three studies do not provide clear information about the setting of the dance intervention (Baptista et al., 2012; Thornberg et al., 2012; Mandelbaum et al., 2016). The review found two studies within a university setting (Aşçi, 2002, 2009).

#### Intervention content and timeframe

Almost half of the studies offer social dance as intervention content (Pinniger et al., 2012, 2013a,b,c; Fonseca et al., 2014; Mandelbaum et al., 2016). Five of them investigate a population with special disorders (Pinniger et al., 2012, 2013a,b,c; Mandelbaum et al., 2016). Other interventions choose step dance (Aşçi, 2002, 2009), belly dance (Baptista et al., 2012), Brazilian folk dance (Soares Costa de Mendonça et al., 2015) and improvisational/creative dance (Thornberg et al., 2012). Two studies offer a program with various dance styles (Stickley et al., 2015; Kosmat and Vranic, 2017). The interventions show different timeframes. The minimum duration is 2 weeks (Pinniger et al., 2013c). The study conducted by Stickley et al. shows the largest duration (30 months), but the attendance rate for inclusion in analysis is set at a minimum of eight individual classes. There was no information available about the general dance training frequency or the required frequency for inclusion in this study (Stickley et al., 2015). All other studies offer dance in one up to four sessions a week. One study lacks clear information about the type of participation, in all the other studies the participation is voluntary (Baptista et al., 2012).

#### Research approach and design

Different research approaches and designs can be found. Eight studies are randomized controlled trials (RCTs) with pre-post-testing (Aşçi, 2002, 2009; Baptista et al., 2012; Pinniger et al., 2012, 2013a,b,c; Kosmat and Vranic, 2017), four of them include follow-up measures (Baptista et al., 2012; Pinniger et al., 2013b,c; Kosmat and Vranic, 2017). Furthermore, Soares Costa de Mendonça et al. use a design with three cluster-randomized experimental groups and one control group (Soares Costa de Mendonça et al., 2015). A study undertaken by Costa Fonseca et al. with a pre- and post-test design does not provide information about a conducted randomization for the experimental and control group (Fonseca et al., 2014). Mandelbaum et al. conduct a pilot study with a pre- and post-test design and two follow-ups, but do not have a control group (Mandelbaum et al., 2016). Stickley et al. use a mixed methods approach combining a post-test quantitative questionnaire with qualitative interviews and discussions (Stickley et al., 2015). Thornberg et al. conduct a qualitative study and analyze interviews and DVD recordings (Thornberg et al., 2012).

#### Investigated aspects of the self and results

The one study, which uses qualitative methodologies, highlights positive development in aspects of the self as main topic of the analysis. The participants report positive changes in terms of self-/body-awareness and self-development (Thornberg et al., 2012).

Different outcomes have been addressed by studies with quantitative methodologies. Three studies investigate body-related perceptions: two studies report a positive effect on physical self-perception (Aşçi, 2002) and satisfaction with physical appearance (Soares Costa de Mendonça et al., 2015). It should be noted that in the study conducted by Soares Costa de Mendonça et al. a positive effect on the satisfaction with physical appearance is found in all active experimental groups (dance, strength training, hydrogymnastics) (Soares Costa de Mendonça et al., 2015). However, the study does not find positive effects on body image perception (Soares Costa de Mendonça et al., 2015). The study results of Fonseca et al. do not show positive effects on body size perception but tendencies that dance can help in gaining an appropriate body perception (Fonseca et al., 2014). Four studies investigate the effects on self-efficacy. An improvement in self-efficacy is stated in one study for the post-test but not for the follow-up measures (Pinniger et al., 2013c). Three studies expose no improvement at the post-test as well as at follow-up (Pinniger et al., 2013b; Mandelbaum et al., 2016; Kosmat and Vranic, 2017). Moreover, three studies investigate self-esteem. Two of them find positive effects on self-esteem for the dance group (Pinniger et al., 2013a; Soares Costa de Mendonça et al., 2015). Again, the study conducted by Soares Costa de Mendonça et al., exposes improvement in self-esteem for all experimental groups (Soares Costa de Mendonça et al., 2015). The third study focusing on self-esteem does not report a positive effect of tango dance (Pinniger et al., 2012). Furthermore, two studies analyze the effect on self-image and self-concept, respectively. The RCT study by Baptista et al. outlines a positive effect on self-image in the dance group, which maintained at follow-up (Baptista et al., 2012). Aşçi investigates self-concept. An overall improvement of self-concept is absent. However, positive effects are reported for physical self and identity dimensions of the self-concept (Aşçi, 2009).

One study choses a mixed methods approach (Stickley et al., 2015). The results of the qualitative analysis indicate a positive impact of dance as the participants report positive development in self-expression, self-efficacy and self-confidence. The quantitative measures support these findings in part as they show positive tendencies in terms of self-expression. However, the study does not provide significance levels.

#### Study quality

The Tables 6, 7 inform about the fulfillment of important quality criteria. The detailed ratings for each quantitative study are presented in Table A1 in Supplementary Material.

Two studies work with qualitative measures. The study conducted by Thornberg et al. fullfills 8 out of 10 criteria of the methodological assessment tool (Thornberg et al., 2012). The study undertaken by Stickley et al. meets half of the criteria (Stickley et al., 2015). Neither study provides a statement locating the researchers culturally or theoretically. Moreover, a clear declaration of the influence of the researcher on the research, and vice versa, is absent.

Twelve studies follow a quantitative approach. The methodological quality of most studies ranges in the middle or upper third of the quality assessment scale, with mean values from 0.25 to 0.75 (Aşçi, 2002, 2009; Baptista et al., 2012; Pinniger et al., 2012, 2013a,b,c; Soares Costa de Mendonça et al., 2015; Mandelbaum et al., 2016; Kosmat and Vranic, 2017). Two studies deviate from the majority as they show mean values of 0.08 (Fonseca et al., 2014) and −0.33 (Stickley et al., 2015). Main reasons for low ratings are small samples as well as missing information about the handling of missing data. Nine studies select their participants in randomized fashion. All studies fullfill the operationalization of concepts and present statements subject to ethical criteria in an appropriate way. Nine studies present results of effect size measures.

## Discussion

This systematic review aimed at giving an overview of studies that investigate the effects of dance interventions on aspects of the participants' self. The results indicate that dance can have positive effects on different aspects of the self in children/adolescents and adults, especially on body-related perceptions. Despite positive findings, evidence for the different aspects is still poor due to the small number of studies on each construct and/or inconsistent findings. The evaluated studies show an overall heterogeneous nature of research designs and methods as well as intervention contents and timeframes. Furthermore, they differ in terms of methodological quality.

### Dance interventions in children and adolescents

#### Effects on the participants' self

In the field of *body-related perceptions* one qualitative study was identified (Jounghwa et al., 2013). The results correspond to theoretical assumptions: dance promotes the ability of self-expression via the body. Apparently, this positive development is associated with an awareness of numerous opportunities for nonverbal expression through different body movements and with an enhancement of positive perceptions about the body (Jounghwa et al., 2013). Studies with quantitative methodologies investigating body-related perceptions report positive effects—with moderate effect size—on body self-concept (Studer-Lüthi and Züger, 2012) or no significant effects on body image but positive tendencies (Caf et al., 1997). One quantitative study reports inconsistent findings concerning body-related perceptions: Blackman et al. do not find improvement of attitudes toward the subject's own body but on the physical subscale of self-concept (Blackman et al., 1988). Overall, these quantitative results rather support the qualitative findings. Furthermore, they support the results found by Burkhardt and Brennan (2012), who identified two studies showing improved body image/-attractiveness and physical self-worth through aerobic dance participation in adolescent females. However, as aerobic dance rather focuses on fitness aspects than on the self-expression via the body, a comparison of their results and the results of this review must be interpreted with caution. It remains unclear if an improvement of body-related perceptions arises from physical activity per se or from the specific activity that involves movement to music. The fact that the study results show inconsistent findings on closely linked constructs reflects the complexity of the outcome field. Furthermore, questionnaires differ concerning their scales and items that may be more or less suitable to capture the impact of dance experiences. Question items at a very general level may not be directly transferable to the dance context. Looking at the studies' quality, the qualitative study fulfills seven out of 10 quality criteria and therefore performs well (Jounghwa et al., 2013). The quantitative studies on this outcome range in the lower (Caf et al., 1997) and middle third (Blackman et al., 1988; Studer-Lüthi and Züger, 2012) of the quality rating scale. Nevertheless, one of them fulfills important quality criteria like quasi-randomization, control and the calculation of effect sizes (Studer-Lüthi and Züger, 2012). Summarizing the results of qualitative and quantitative measures, evidence for effects of dance on body-related perceptions is low. Main reasons are the small number of studies and their overall moderate quality. However, the predominant positive impact supports theoretical explanations, since dance is characterized as an activity with an internal orientation including the awareness of the own body in motion (Ullmann, 2001; Gardner et al., 2008).

The review found inconsistent results on effects of dance on *self-esteem* in young people and thus poor evidence for this outcome. All studies use quantitative methodologies. Two studies report positive effects (Connolly et al., 2011) or no significant effects but positive tendencies (Backe and Graefe, 2004) and two studies show no (Blackman et al., 1988) or negative effects (Jago et al., 2015). Nevertheless, another review points out the positive impact of creative interventions on the self-esteem of children and young people, even if evidence is weak (Bungay and Vella-Burrows, 2013). The review analyses interventions with different forms of performing arts (e.g., music, singing, drama/theater, visual arts, dance) as content. This indicates that dance interventions with a focus on the inherent creative aspects of dance may have the potential to improve self-esteem. In this review, the detected discrepancies of the study findings in terms of self-esteem may be the result of differences concerning the dance interventions. For example, the interventions differ in terms of dance content (only one study shows an emphasis on creative techniques; Backe and Graefe, 2004) and duration. The quality of studies reporting positive effects ranges in the lower (Backe and Graefe, 2004) and middle third (Connolly et al., 2011) of the rating scale. The studies reporting no or negative effects perform better with ratings in the middle (Blackman et al., 1988) and upper third (Jago et al., 2015) of the rating scale. However, the negative results of the study with the best quality must be interpret with caution due to attendance problems (Jago et al., 2015). Ekeland et al. review the effects of exercise on the self-esteem of children and young people and point out methodological shortcomings, too (Ekeland et al., 2005). Over half of their 25 included studies show a high risk of bias. Hence, achieving high study quality seems to be challenging for various activity intervention studies promoting self-esteem in young people.

Evidence for the effects of dance interventions with young people on *self-concept, self-trust, perceptions of the subjects' own abilities* and *self-efficacy* is poor, as the review identified only one or two studies that report results concerning each of these constructs, respectively. However, the positive findings of qualitative studies in terms of self-trust and the perceptions of the subjects' own abilities (Zitomer and Reid, 2011; Duberg et al., 2016) lie close to adjacent research fields: Bungay and Vella-Burrows review the effects of participating in creative activities on health and well-being of children and young people (Bungay and Vella-Burrows, 2013). An increased confidence is the most commonly reported outcome in all studies they review. Moreover, the mentioned studies fulfill all (Duberg et al., 2016) or at least 9 out of 10 (Zitomer and Reid, 2011) quality criteria and thus can serve as an example of qualitative research of high quality in the field.

#### Dance intervention conditions

Almost half of the samples consist of *children and adolescents with special disorders*. Out of the five studies, four report positive findings: studies with qualitative methodologies point out positive changes in terms of self-trust (Duberg et al., 2016) and perceptions of dance ability (Zitomer and Reid, 2011) as main topics of their analysis; studies with quantitative methodologies outline effects in subscales of body image (Caf et al., 1997) and no significant effects but positive tendencies in self-esteem (Backe and Graefe, 2004). Three out of five respective studies (Roswal et al., 1988; Caf et al., 1997; Backe and Graefe, 2004) state that they choose *creative dance* as intervention content. A closer look at the described intervention lessons shows that the two other interventions with participants with special disorders work with creative techniques as well. They offer processes of movement exploration/improvisation (Zitomer and Reid, 2011; Duberg et al., 2016) and cooperative group works giving space to different executive modes based on different physical capacities and interests (Zitomer and Reid, 2011). Creative dance is oriented toward the movement opportunities of the individual person. It pursues exploring movement possibilities and the solving of movement oriented tasks to enrich self-expression (Laban, 1948). Due to the student-centered and non-normed way of understanding and teaching dance, students with certain limitations can possibly experience success and self-efficacy. However, two of these studies—both mixed methods studies—show substantial methodological shortcomings (Caf et al., 1997; Backe and Graefe, 2004). Hence, creative dance may be an appropriate activity for children and adolescent with disorders to enhance certain aspects' of the self, but evidence is still poor.

Moreover, two *creative dance* interventions with a sample without special disorders report positive effects (Studer-Lüthi and Züger, 2012) or positive findings, revealed from a qualitative analysis, (Jounghwa et al., 2013) regarding aspects of the participants' self as well. Therefore, creative dance may also be an appropriate activity intervention to strengthen personal aspects of children and adolescents without special disorders. As the review identified only two respective studies, evidence is poor as well.

The reviewed intervention studies show different *timeframes*. Regarding the study results, there seems to be no relation between a certain duration or frequency of dance interventions and their efficacy. This assumption is in accord with the findings of a systematic review in an adjacent research field (Ekeland et al., 2005): Ekeland et al. report that there are few or no differences concerning the results when they excluded the studies with an intervention duration of less than 10 weeks from their analysis. On the contrary, dance interventions that aim to improve physical fitness of young people seem to require a certain minimum timeframe: all respective studies in the review of Burkhardt and Brennan lasted at least 8 weeks and were realized in at least three 45-minute sessions per week (Burkhardt and Brennan, 2012). This appears conclusive, as physical adaptation requires a certain duration and intensity. Positive development in aspects of the self may potentially arise faster. Hence, there seem to be more important influencing factors than the intervention timeframe that may have impact on personal constructs. Especially the influence of the teacher and methodical-didactical concepts may play an important role. However, the only identified study of a comparative nature does not find positive effects on self-concept in both programs (Roswal et al., 1988).

Eight of the studies with children and adolescents take place in a *school setting*. Most studies conducted in this setting report positive effects in terms of body-related perceptions, self-esteem or self-efficacy determined by their quantitative measures (Blackman et al., 1988; Caf et al., 1997; Connolly et al., 2011; Romero, 2012; Studer-Lüthi and Züger, 2012) or point out positive changes in the ability of self-expression and the perceptions about the body as main topic of their qualitative analysis (Jounghwa et al., 2013). These positive results indicate that school could be a valuable setting for dance interventions. However, this review discovered two critical issues of dance interventions in the school setting. Firstly, we found different participation types. In four studies the participation was purely voluntary. Three of them report positive findings regarding aspects of the self (Blackman et al., 1988; Caf et al., 1997; Romero, 2012). Three studies show a mandatory participation and two of them report positive findings (Studer-Lüthi and Züger, 2012; Jounghwa et al., 2013). One quantitative study investigates a sample with voluntary as well as mandatory participants and outlines positive effects on self-esteem (Connolly et al., 2011). They report that different participation types may have had an impact on intrinsic motivational levels, but it was not possible to separate these two groups for examining group differences. Moreover, the only study with an overall quality in the upper third of the rating scale has to deal with attendance problems; it was organized as an after-school dance program with voluntary participation (Jago et al., 2015). The critical issue of adherence is discussed by adjacent research fields such as the investigation of the effects of yoga interventions. Büssing et al. mention that the potentially beneficial impact of yoga can be limited by low adherence rates (Büssing et al., 2012). In summary, we are not able to draw a conclusion from the empirical data whether a certain participation type is associated with more positive results. The design of comparative studies could be an approach to answer this question. However, the problems in terms of attendance rates may influence the findings of comparative studies, too. Secondly, we discovered critical issues concerning the study design and quality. None of the studies in a school setting is organized as a randomized controlled trial. We identified only three studies with quasi or cluster randomized groups (Roswal et al., 1988; Studer-Lüthi and Züger, 2012; Jago et al., 2015). Moreover, three out of seven studies with quantitative measures lack a control group (Roswal et al., 1988; Connolly et al., 2011; Romero, 2012). The samples consist of 40 participants on average (excluding one study with an exceptional sample size of *N* = 508). Overall, study quality of studies with quantitative measures ranges mainly in the lower and middle third of the rating scale. The review found no single quantitative study with a high study quality in a school setting and an applicable assessment of the effects of dance on aspects of the self (Jago et al., 2015)^4^. Looking at the studies with a qualitative approach, we found one study that meets seven out of ten quality criteria (Jounghwa et al., 2013) and one study with only two criteria fulfilled (Caf et al., 1997). In view of these results, achieving high study quality seems to be challenging in particular in a school setting. Studer-Luethi and Zueger as well as Burkhardt and Brennan mention that those studies have to deal with certain framework conditions that may place some limits for research (Burkhardt and Brennan, 2012; Studer-Lüthi and Züger, 2012). Nevertheless, school could function as a valuable setting due to the relative heterogeneity of the potential sample. All potential children and adolescents can be reached. Moreover, it offers the possibility of mandatory participation in a certain intervention, for example through an embedding in physical education lessons. Given the fact that national curricula demand the promotion of constructs like the students' self-confidence, self-awareness and self-competence^5^, additional high-quality research on dance interventions in the school setting is needed to examine the assumption that dance strengthens personal aspects. With regard to *study design and quality* in different settings, only three studies are quasi or cluster randomized controlled trials with pre- and post-testing (Roswal et al., 1988; Studer-Lüthi and Züger, 2012; Jago et al., 2015). Authors of two other systematic reviews concerning performing arts and recreational dance interventions with young people (Daykin et al., 2008; Burkhardt and Brennan, 2012) also report an underrepresentation of randomized controlled trials. Furthermore, only three out of eight studies with quantitative measures have a control group (Caf et al., 1997; Studer-Lüthi and Züger, 2012; Jago et al., 2015). The reviewed studies of Daykin et al. perform little better (Daykin et al., 2008): six out of nine quantitative studies are identified as controlled studies; three of them are identified as randomized controlled trials. One possible reason for the higher rate of controlled and randomized studies may be that they included only interventions outside the usual curriculum. We found only one study with follow-up measures (Jago et al., 2015). This is similar to the findings of Ekeland et al. as their reviewed exercise intervention studies with a sample of children or young people lack follow-up measures (Ekeland et al., 2005). Thus, the implementation of rigorous and complex research designs seems to be a general challenge in exercise and dance intervention studies with a sample of young people, which aim to promote aspects of the self. None of the controlled studies in this review has an active control group. Therefore, the studies cannot answer the question of whether the intervention focus on dance is the reason for the reported effects. Additionally, only one out of five studies with qualitative measures clearly addresses the influence of the researcher on the research and vice versa (see Table 6, Q7). This seems to be important in particular when the role of the researcher and the teacher coincide. These issues are also emphasized by Daykin et al. as most of their reviewed performing arts intervention studies do not include a reflexive discussion of the relationships between researcher and participants (Daykin et al., 2008).

### Dance interventions in adults

#### Effects on the participants' self

Overall, the results indicate that dance interventions can have positive impact on *body-related perceptions* of adults: a qualitative study points out that the participants benefit in terms of body-awareness (Thornberg et al., 2012). The fact that quantitative studies report positive effects—with small or medium effect sizes—on physical self-perception (Aşçi, 2002, 2009) and positive tendencies in body size perception support this finding (Fonseca et al., 2014), while one study outlines inconsistent results (Soares Costa de Mendonça et al., 2015). The positive trend is comparable to the impact of dance on body-related perceptions in children and adolescents, even though the studies differ with regard to their intervention content: while we found a high proportion of creative dance techniques in studies for young people, the studies with an adult sample offer step dance, social dance and folk dance with a focus on deductive methods. One possible explanation could be that dance—regardless of style—may strengthen body-related perceptions of participants of different age groups. However, it could also be possible that different age populations benefit from different intervention contents. As the review lacked a study with an adult sample, which compares different dance contents, future research may address this question. Nevertheless, study quality of studies on this outcome field ranges in the middle and upper third for quantitative studies and the qualitative study fulfills eight out ten quality criteria. Therefore, the studies perform better concerning methodological issues in comparison to studies with children/adolescents. In summary, the positive findings on this outcome show that dance may be an alternative intervention content to exercise in studies that aim to promote body-related perceptions in adult populations. Exercise interventions have already been shown to improve physical self-worth and further body-related perceptions in middle aged and older adults (McAuley et al., 1997; Li et al., 2002).

The five studies examining effects on *self-efficacy* show inconsistent results. One mixed methods study outlines positive development in self-efficacy as the key topic of the discussions and interviews (Stickley et al., 2015). Three out of four quantitative studies reveal no improvement (Pinniger et al., 2013b; Mandelbaum et al., 2016; Kosmat and Vranic, 2017), even though the one study finding positive results reports high effect sizes (Pinniger et al., 2013c). An analysis of the results in view of the intervention characteristics (e.g., population, timeframe, dance content) reveals no explanation for the reasons of these discrepancies: the studies reporting positive results show different characteristics as well as the studies that outline no improvement. An adjacent summary of reviews concerning the effects of yoga interventions on mental and physical health of adults reports positive effects of yoga on self-efficacy (Büssing et al., 2012). Obviously, there exist clear differences between yoga and dance. Nevertheless, yoga as well as dance are characterized through an inner orientation during movement execution and thus may both have a similar impact on aspects of the self. However, the inconsistent results of the studies in this review cannot support this assumption.

For the remaining constructs, the review identified only a small number of studies and therefore evidence is poor. Moreover, the studies that investigate *self-esteem* or *self-concept/self-image* (all quantitative) report inconsistent results (Aşçi, 2009; Baptista et al., 2012; Pinniger et al., 2012, 2013a; Soares Costa de Mendonça et al., 2015), which is similar to the studies with children and adolescents for self-esteem. Nevertheless, two out of three studies that investigate self-esteem find positive effects with high effect sizes (Pinniger et al., 2013a; Soares Costa de Mendonça et al., 2015). Two qualitative studies point out findings in terms of *self-development, self-expression* and *self-confidence*. They consistently report a positive impact of dance interventions on the mentioned constructs. These first results could form the basis for future research on these topics.

#### Dance intervention conditions

The *samples* of eight out of the thirteen reviewed studies present a wide age range (Baptista et al., 2012; Pinniger et al., 2012, 2013b,c; Fonseca et al., 2014; Soares Costa de Mendonça et al., 2015; Stickley et al., 2015; Mandelbaum et al., 2016). Potential disparities concerning the impact on participants of different age groups cannot be captured, as the studies do not compare subgroups (e.g., younger vs. middle-aged vs. older adults). Designing comparative studies that offer the same specific dance program to adults of different age groups would be a possible approach to this question. The samples consist of about 60 participants on average (excluding one study with an exceptional sample size of *N* = 330). Hence, these studies show little larger sample sizes than those with a population of children/adolescents. However, with respect to the low generalizability of their results, studies with larger sample sizes are desirable for future research. This is also claimed by other authors of a systematic review in the field (Hwang and Braun, 2015).

In contrast to dance interventions for children and adolescents almost half of the studies chose social dance as *content* (Pinniger et al., 2012, 2013a,b,c; Fonseca et al., 2014; Mandelbaum et al., 2016). According to Pinniger et al., partnered dance requires an awareness of one's own body as well as the partner's body (Pinniger et al., 2012). Moreover, the emphasis of choosing social dance as content may reflect the general meaning of partnered dance for adult generations in comparison to young people. Social dance seems to be especially chosen for adult participants, though the study results (all quantitative studies) are inconsistent and therefore show poor evidence: three studies do not find improvement in self-esteem and self-efficacy (Pinniger et al., 2012, 2013b; Mandelbaum et al., 2016), while three studies report positive effects—with high effect sizes—on the mentioned constructs (Pinniger et al., 2013a,c) or no significant effects but positive tendencies on body size perception (Fonseca et al., 2014). It should be noted that these discrepancies may also be less a result of the specific intervention but of an apparent difficulty in measuring these constructs with quantitative methodologies. Moreover, five out of the six studies investigate a sample with special disorders. The differences concerning the population may be another reason for the inconsistent results. In conclusion, the reviewed studies cannot answer the question whether partnered dance can strengthen aspects of the self for adult populations with or without disorders.

The reviewed studies show different intervention *timeframes*. With respect to the results, the frequency of the dance sessions for adults seems to play a role especially for short- and medium-duration programs up to 12 weeks. A higher frequency may contribute to more positive effects. For interventions with a duration of at least 16 weeks the frequency seems to play less of a role regarding their effectiveness. In contrast to the reviewed interventions with children/adolescents, frequency is detected as relevant for the effectiveness of dance interventions with adults. However, the study with the longest duration (30 months) does not provide precise information concerning the dance training frequency (Stickley et al., 2015). Due to methodological shortcomings, the study cannot contribute to the assessment of the effects of participating in a long-term dance program on aspects of the self. As dance interventions with a long duration are rare, future research should seek the effects of long-term in comparison to short-term programs.

With regard to *study design and quality*, we found considerably more randomized controlled trials for adults than for children and adolescents. Eight out of twelve studies with *quantitative methodologies* are randomized controlled trials (Aşçi, 2002, 2009; Baptista et al., 2012; Pinniger et al., 2012, 2013a,b,c; Kosmat and Vranic, 2017). Moreover, five studies (Baptista et al., 2012; Pinniger et al., 2013b,c; Mandelbaum et al., 2016; Kosmat and Vranic, 2017) conduct follow-up measures, which was rare for studies with a sample of young people. The quality of these studies ranges in the middle and upper third of the rating scale. Only two studies with quantitative measures lack a control group (Stickley et al., 2015; Mandelbaum et al., 2016). The fact that nine out of 12 studies with quantitative measures calculate effect sizes enhances the credibility of the study results, too. However, the studies differ in terms of outcomes, measurement tools and intervention frameworks. Hence, at the current state of research, there is still a too small number of studies of comparable frameworks investigating the same outcome to perform a meta-analysis. Most of the studies have non-active control groups, as already ascertained for studies with young people. This issue is also noted by Hwang and Braun who highlight the effectiveness of dance interventions to improve older adults' physical health (Hwang and Braun, 2015). The consideration of the activity level of the control groups seems to be one respective issue for studies in adults. Nevertheless, with respect to the overall quality and important quality criteria like randomization, control and effect size measures, quantitative studies with adult samples perform better than those with samples of young people.

The review identified only two studies with adults with *qualitative measures* (Thornberg et al., 2012; Stickley et al., 2015) while one of them fulfills only half of the quality criteria (Stickley et al., 2015). Thus, we are not able to draw a conclusion about the potential benefits of qualitative approaches in this research field from the empirical data. However, as knowledge and evidence on effects of dance interventions are scarce to date, a more open approach with qualitative methodologies may discover impact fields that are rather unknown. As determined for studies with a sample of young people, the two studies with adults show problems in giving a clear declaration of the influence of the researcher on the research, and vice versa (see Table 6, Q7). The importance of the teacher as an influencing factor in a teaching context should be emphasized (e.g., teaching/feedback style). Additionally, both studies lack a statement locating the researchers culturally and theoretically (e.g., information about vocational training/focus) (see Table 6, Q6). We recommend taking such a statement into account, as it can give more insight into the background of the dance intervention, which plays an important role for its development.

### General aspects

In the following, we discuss issues that seem to be relevant when planning dance intervention studies regardless of their population. These issues may give impulses for future studies in the field.

#### Investigated aspects of the self

The studies focus on *different aspects of the self*. The review yielded only a little number of studies focusing on exactly the same outcome, though most of them are closely related. For example, quantitative studies investigating body-related perceptions measure the following outcomes: attitudes toward the subject's own body (Blackman et al., 1988), body image (Caf et al., 1997), body self-concept (Studer-Lüthi and Züger, 2012), physical self-perception (Aşçi, 2002), body size perception (Fonseca et al., 2014), satisfaction with physical appearance and body image perception (Soares Costa de Mendonça et al., 2015). This also entails that different measurement instruments are used, which may lead to discrepancies concerning study results. Furthermore, the selection of an appropriate outcome is connected with the range of existing validated questionnaires for quantitative measurements. Roswal et al. address the question of appropriateness of the existing questionnaires in the dance context (Roswal et al., 1988). They highly recommend the development of dance-specific tools for future research. Progress in the field of designing and validating suitable measurement instruments for quantitative research is expected from a research group from Germany^6^. They work on the development of a measurement instrument concerning the dance-specific self-concept for children at the age of 8 to 12. More dance specific measurement tools for further population groups are desirable.

Moreover, some quantitative studies choose to measure the effect of dance interventions on the *self-concept* or rather *self-image* of the participants. None of the studies that investigate self-concept report an improvement of the overall construct (Blackman et al., 1988; Roswal et al., 1988; Aşçi, 2009). Only one study finds a positive effect on self-image (Baptista et al., 2012). Looking at these study results, dance interventions do not seem to be able to improve self-concept. However, the review of Burkhardt and Brennan indicates that dance interventions (including fitness dance interventions, e.g., aerobic dance) may improve self-concept, but with the addition that these assumptions are based on very limited evidence (Burkhardt and Brennan, 2012). Stein—referring to Bracken et al.—reports that intervention studies often fail to increase self-concept (Stein, 2011). She emphasizes the insufficient sensitivity of global self-concept to specific treatments. Self-concept or self-image are constructs of a superordinate level in comparison to subordinated, more specific constructs like the physical self-concept or even more precisely the self-perception of the subject's own dance ability (Marsh and Hattie, 2011). Outcomes at a very general level may be inappropriate for most of the determined timeframes of the reviewed interventions due to their relative stability over time (Marsh and Hattie, 2011). Therefore, future research may ask for the effects of dance in a more specific manner. In relation to the inherent orientation of dance, body awareness—among other things—may be a specific relevant construct. The assessment of potential transfer effects on connected constructs like the physical self-concept may be of interest, too. Connolly et al. also recommend the investigation of constructs that are connected to the uniqueness of dance (Connolly et al., 2011). They point out the inherent creativity in dance, the forum it presents for task- and problem-solving as well as the interactions of music and movement.

#### Presentation of intervention contents and conditions

The reviewed interventions choose different dance contents. In several cases, we missed detailed information about the intervention content. Some of the reviewed studies do not provide precise information concerning relevant issues in a teaching context, such as teachers' methodical-didactical concepts or lesson contents (see Table 5). Furthermore, the studies choose different dance styles for their intervention. In several studies, the reasons for the choice of a certain dance form is explained (Roswal et al., 1988; Caf et al., 1997; Backe and Graefe, 2004; Zitomer and Reid, 2011; Pinniger et al., 2012, 2013a,b,c; Romero, 2012; Jounghwa et al., 2013; Fonseca et al., 2014; Mandelbaum et al., 2016). However, some studies do not give reasons for their choice of a specific dance style or do not even chose a specific style. Depending on dance form or, even more important, the methodical-didactical way of teaching, there may be more or fewer opportunities to advance participants in aspects of the self, e.g., in terms of self-expression. In such a complex context, diverse factors may influence the study results and should therefore be reported as effectively as possible: information about the teacher, educational approach/focus, methodical-didactical concepts (e.g., normed or non-normed), feedback manner, social setting, participation type or dance style—among others—may be relevant for interpreting reported results. The poor reporting of dance intervention details is also addressed in the review conducted by Burkhardt and Brennan (Burkhardt and Brennan, 2012). They refer to the limited reproducibility of the interventions, when studies do not provide details. Moreover, future research should choose the intervention content in relation to the aim of the intervention. In this case, one has to ask which dance form has the most potential to obtain positive effects on certain psychological constructs. Schmais and White emphasize that dance forms differ concerning their organization and structure, which may place limits on the dance experience (Schmais and White, 1986). This assumption counts for the methodical-didactical concept of teaching dance as well, since different dance styles can be taught in different ways (e.g., implementation of creative teaching methods vs. standardized training of dance techniques). We recommend that future studies open the “black-box” by introducing the intervention content, timeframes and other framework conditions in sufficient detail. Presenting the full details of the intervention components in a separate publication can be an approach to consider the complexity of a dance intervention. Publishing an intervention mapping protocol or the use of checklists for a better reporting of the intervention (e.g., TIDieR guidelines) could be a possibility for future research to present intervention design and contents more precisely (Hoffmann et al., 2014).

#### Research approaches and methods

We found an emphasis on studies using quantitative measures, especially within adult samples. The review yielded only one study with an adult sample and a purely qualitative approach (Thornberg et al., 2012). Daykin et al. emphasize that qualitative methods have already been successfully used in performing arts interventions (e.g., action research, grounded theory, ethnography) (Daykin et al., 2008). Regarding the holistic nature of dance, it seems questionable to choose specific outcomes to be measured (Duberg et al., 2016). However, for a quantitative survey with standardized questionnaires a certain outcome must be focused. Daykin et al. state that a qualitative approach may be more suitable to address impact and process issues in relation to performing arts for health (Daykin et al., 2008). Choosing a research approach and design suitable to the study's objective is seen as a challenge of complex interventions such as performing arts. This statement counts for teaching contexts as well and is highly relevant for future dance intervention studies.

### Strengths and limitations

The conducted review has certain strengths but also limitations. The review was accomplished according to the PRISMA guidelines to ensure information about the review process and analyses were sufficiently described. Furthermore, we chose relatively open inclusion criteria to identify a wide range of studies in the field. This was due to the fact that the aim was to give an overview of the implemented study approaches, designs and measurements as well as the investigated settings and populations. For the development of future intervention studies in the field, it is important to know if research focused on aspects such as investigating a certain population group in particular or on using quantitative methodologies. However, this open strategy led to a limitation of the comparability of the assessed studies. Additionally, several databases were searched. We arranged the independent screening and study selection process as well as the methodological quality assessment by two reviewers. The CCEERC Quantitative Research Assessment Tool^7^ as well as the JBI Checklist for Qualitative research (Lockwood et al., 2015) were assessed to rate the studies' quality. The search was restricted to journal articles in English or German. We excluded reports and gray literature. Therefore, we cannot rule out the existence of other relevant studies in the field. Due to the screening of titles and abstracts, the review team was dependent on a certain quality of the abstracts to identify relevant studies. Relevant literature could have been lost because of imprecise abstracts. The review relates to educational and teaching contexts and therefore is affected by the critical debate of systematic reviews in an educational context. The reviewers as well as the readers have to be aware of the limitations of a systematic review. In accordance to Andrews, this systematic review must be seen as an attempt to minimize bias (Andrews, 2005). We do not claim to eliminate bias or to present a complete, objective version of the current state of research. However, we chose a systematic approach due to the advantages mentioned in the methods chapter. In using the PRISMA checklists and recommendations we tried to consider the demand for transparency, validity and reproducibility.

## Conclusion

This systematic review aimed at giving an overview of studies that investigate the effects of dance interventions on aspects of the participants' self. Overall, the review of 24 included studies indicates that a dance intervention can advance the participants in different aspects of the self. Studies with qualitative methodologies find that children/adolescents benefit in body-related perceptions, self-trust, self-esteem, self-expression, and the perception of dance-abilities. Moreover, dance interventions may strengthen self-expression, self-efficacy, self-/body-awareness, self-development, and self-confidence in adults. Studies with quantitative methodologies point out improvement especially for body-related perceptions in children/adolescents as well as in adults. The review revealed heterogeneous results concerning self-esteem and self-efficacy. Furthermore, the evaluated studies show an overall heterogeneous nature of populations, intervention contents, timeframes, outcomes, research methods and study quality. Despite positive findings, evidence for each of the aspects is still poor due to the small number of studies on each construct, inconsistent findings or methodological shortcomings. There is a need for more high-quality studies in the field in order to further evaluate the findings and to gain a deeper understanding of the possible benefits of dance interventions in different populations.

The following recommendations for future intervention studies in the field can be derived from the review: large samples and intervention timeframes are desirable. Especially for quantitative studies with a sample of children and adolescents, the implementation of randomized controlled and quasi-experimental trials with active control groups is detected as an issue for future research. Follow-up measures are needed to examine whether the effects of the dance interventions persist over time. However, it often seems to be difficult to conduct these study designs with complex measurements in school settings. Additionally, future research may consider using mixed methods or qualitative study approaches. The implementation of comparative studies may address the questions of whether different participation types or methodical-didactical concepts influence study results. As we have to deal with a complexity of the respective construct as well as the interventions themselves, future studies should be designed carefully and with regard to methodological rigor. Researchers should reflect their study approach/design, the intervention content as well as the constructs to be investigated considering the specificity of dance. The design and validation of appropriate measurement instruments in the dance context is desirable. The presentation of the intervention contents, timeframes and other framework conditions in sufficient detail would be beneficial to enable reproducibility of the intervention as well as transparency of the interpretation of the reported results.

## Author contributions

TS, SS, and FM conceived and designed the study. TS and CO performed the literature search and selection process. TS, SS, and CO performed the methodological quality assessment. TS analyzed the data and wrote most of the paper with substantial contributions from SS and FM.

### Conflict of interest statement

The authors declare that the research was conducted in the absence of any commercial or financial relationships that could be construed as a potential conflict of interest.

## Footnotes

^1^International prospective register of systematic reviews. https://www.crd.york.ac.uk/PROSPERO/.

^2^Quantitative Research Assessment Tool provided by the Child Care and Early Education Research Connections (CCEERC). Available online at: https://www.researchconnections.org/content/childcare/understand/research-quality.html.

^3^The Cochrane Collaboration. Cochrane Handbook for Systematic Reviews of Interventions. Version 5.1.0. Available online at: http://handbook-5-1.cochrane.org/.

^4^Jago et al. state that it is not possible to use the data from their project to assess the effects of attending dance programmes on self-esteem or other health outcomes. Only one-third of the intervention participants met the pre-set attendance criteria.

^5^Staatsinstitut für Schulqualität und Bildungsforschung München. Lehrplan für das Gymnasium in Bayern. Fachprofil Sport. Retrieved from http://www.isb-gym8-lehrplan.de/contentserv/3.1.neu/g8.de/index.php?StoryID=26406; Australian Curriculum Assessment and Reporting Authority. Introduction of the Australian F-10 curriculum. Retrieved from https://www.australiancurriculum.edu.au/f-10-curriculum/general-capabilities/personal-and-social-capability; Ministry of Education Ontario. The Ontario Curriculum Grades 1 to 8. Health and Physical Education. Retrieved from http://www.edu.gov.on.ca/eng/curriculum/elementary/health1to8.pdf.

^6^Giegerich, P. (2017). *BMBF-Förderung für die Erforschung von Tanz als Mittel kultureller Bildung*. Available online at: https://idw-online.de/de/news670978.

^7^Quantitative Research Assessment Tool provided by the Child Care and Early Education Research Connections (CCEERC). Available online at: https://www.researchconnections.org/content/childcare/understand/research-quality.html.
